# CYP4F2 (rs2108622) Gene Polymorphism Association with Age-Related Macular Degeneration

**DOI:** 10.1155/2016/3917916

**Published:** 2016-08-29

**Authors:** Ruta Sakiene, Alvita Vilkeviciute, Loresa Kriauciuniene, Vilma Jurate Balciuniene, Dovile Buteikiene, Goda Miniauskiene, Rasa Liutkeviciene

**Affiliations:** ^1^Lithuanian University of Health Sciences, Medical Academy, Eiveniu 2, LT-50009 Kaunas, Lithuania; ^2^Neuroscience Institute, Lithuanian University of Health Sciences, Medical Academy, Eiveniu 2, LT-50009 Kaunas, Lithuania; ^3^Department of Ophthalmology, Lithuanian University of Health Sciences, Medical Academy, Eiveniu 2, LT-50009 Kaunas, Lithuania

## Abstract

*Background*. Age-related macular degeneration is the leading cause of blindness in elderly individuals where aetiology and pathophysiology of age-related macular degeneration are not absolutely clear.* Purpose*. To determine the frequency of the genotype of rs2108622 in patients with early and exudative age-related macular degeneration.* Methods*. The study enrolled 190 patients with early age-related macular degeneration, 181 patients with exudative age-related macular degeneration (eAMD), and a random sample of 210 subjects from the general population (control group). The genotyping of rs2108622 was carried out using the real-time polymerase chain reaction method.* Results*. The analysis of rs2108622 gene polymorphism did not reveal any differences in the distribution of C/C, C/T, and T/T genotypes between the early AMD group, the eAMD group, and the control group. The* CYP4F2* (1347C>T)* T/T* genotype was more frequent in males with eAMD compared to females (10.2% versus 0.8%; *p* = 0.0052); also* T/T* genotype was less frequently present in eAMD females compared to healthy control females (0.8% versus 6.2%; *p* = 0.027).* Conclusion*. Rs2108622 gene polymorphism had no predominant effect on the development of early AMD and eAMD. The* T/T* genotype was more frequent in males with eAMD compared to females and less frequently present in eAMD females compared to healthy females.

## 1. Introduction

Age-related macular degeneration (AMD) is a progressive neurodegenerative disease and the leading cause of irreversible blindness among individuals aged 65 and older, particularly in western countries [1]. With the ageing population, the number of people with AMD is estimated to increase by approximately 50% by the year 2020 and the burden of this disease is set to grow [2]. Macular degenerative lesions are manifested by drusen formation, retinal pigment epithelium (RPE) changes, retinal pigment epithelium and choroid capillary layer, Bruch*ʼ*s membrane lesion, geographic atrophy of the central fovea, exudative AMD with choroidal neovascularization, retinal pigmentary epithelium detachment, or submacular disciform scarring changes. The pathological hallmark of the disease is amorphous deposits of protein and lipid, termed drusen [3]. Drusen—colloid material (lipids, phospholipids, and collagen) excrescences, similar to hyaline—accumulate in the retina, in Bruch's membrane underlying the retinal pigment epithelium. This process is associated with retinal pigment epithelium and progressive degeneration of photoreceptors [4]. Drusen disturb oxygenous metabolism and determine the degeneration of photoreceptors, while visual function impairment is associated with the quantity of damaged photoreceptors. In the fovea, where there is the largest quantity of photoreceptors, cones dominate, whereas the parafoveal region, where rods dominate, surrounds the fovea. In the early stages, photoreceptors are mostly damaged in the parafovea.

AMD is a complex disease to which many factors contribute: body ageing together with pathological changes, such as pathogenic oxidative stress, inflammatory processes, changes of the extracellular matrix, biological activity changes in the retinal pigment epithelium, and genetic factors—all of them are important in the pathogenesis of this disease [5]. With advancing age, there is a deposit of lipid particles in a normal Bruch's membrane leading to the creation of a lipid wall, external to the RPE basal lamina, impairing nutrient exchange between the choriocapillaris and the RPE and compromising retinal function [6, 7]. The observation that the location of the lipid wall is the same as and precedes the basal linear deposits and drusen suggests its contribution to drusen formation [8]. Indeed, lipids (both esterified and unesterified cholesterol and phosphatidylcholine) represent at most less than fifty percent of the volume of drusen [9]. Koskela et al. state that the accumulation of oxidized lipids seems to play a pivotal role in the development of AMD [10]. For instance,* CYP4F2* is involved in the production of 20-hydroxyeicosatetraenoic acid (20-HETE), a molecule that is proinflammatory and can induce hyperlipidemia [11, 12].* CYP4F2* is also an important endobiotic metabolizing enzyme involved in the metabolism of fatty acids such as arachidonic acid, medium and very long polyunsaturated fatty acids, eicosanoids such as leukotriene B4 (LTB4), prostaglandins, and lipoxins implicating its importance in maintaining liver polyunsaturated fatty acids (PUFA) levels and inflammatory status [13]. The polymorphism (rs2108622, V433M) in the CYP4F2 gene with a valine to methionine substitution at amino acid 433 was found to be associated with changes in all these processes and, for instance, Fava et al. [14] found that CYP4F2 M433 (V433M) carriers had significantly higher levels of waist, triglycerides, blood pressure (BP), and a composite sum of metabolic syndrome (MetS) phenotypes (MetS score) beside lower high density lipoprotein (HDL) cholesterol with respect to V-homozygotes.

Knowing that the main pathological changes of age-related macular degeneration are drusen, which include about 40% lipids, the attempts to find a relation between age-related macular degeneration and the gene rs2108622 controlling lipid metabolism have been made.

## 2. Materials and Methods

Permission to undertake the study was obtained from the Ethics Committee for Biomedical Research. The study was conducted in the Lithuanian University of Health Sciences (LUHS), Neuroscience Institute, Ophthalmology laboratory (Number BE-2-/13).

The study included patients (*n* = 190) with early age-related macular degeneration and patients (*n* = 181) with exudative age-related macular degeneration and a random sample of the population *n* = 210 (control group).

### 2.1. Control Group Formation

The control group consisted of subjects who had no ophthalmologic pathology on examination and who agreed to take part in this study. The control group involved 210 subjects, who matched the early AMD and eAMD group structure according to their age and gender. Thus it comprised 161 women and 49 men with their ages ranging from 50 to 90 years.

The control group was created by taking into consideration the distribution of age and gender in the early AMD and eAMD group (Table 1). Therefore, the medians of the patient age of the control group and the early and eAMD group did not differ statistically significantly (NS).

During analysis of data, study subjects were grouped according to their age (younger than 65 years and 65 years and older).

### 2.2. Ophthalmological Evaluation

All study subjects were evaluated by slit-lamp biomicroscopy to assess corneal and lenticular transparency. Classification and grading of lens opacities were performed according to the Lens Opacities Classification System III. Best corrected visual acuity (BCVA) was measured using the standard procedure adapted for the Age-Related Eye Disease Study (AREDS) at a 5-meter distance from the chart (letters on the ETDRS logMAR chart), for subjects with sufficiently reduced vision, at 1 meter. At each examination, intraocular pressure was measured. Pupils were dilated with tropicamide 1%, after which fundoscopy, using slit-lamp biomicroscopy with a double aspheric lens of +78 diopters, was performed. For detailed analysis of the macula, colour fundus photographs of the macula, centred at 30° to the fovea, were obtained with a Visucam NM Digital camera (Carl Zeiss Meditec AG, Germany).

The classification system of AMD formulated by the Age-Related Eye Disease Study [15] was used: early AMD consisted of a combination of multiple small drusen and several intermediate drusen (63–124 *μ*m in diameter) or retinal pigment epithelial abnormalities; intermediate AMD was characterized by the presence of extensive intermediate drusen and at least one large drusen (≥125 *μ*m in diameter) or geographic atrophy (GA) not involving the centre of the fovea; and advanced AMD was characterized by GA involving the fovea and/or any of the features of neovascular AMD [16]. Early AMD and eAMD were diagnosed by two ophthalmologists. Simultaneous spectral-domain optical coherence tomography (SD OCT; 870 nm, 40 000 A-scans/sec) and/or fluorescein angiography (FA) were performed for all AMD patients. SD OCT was carried out with Nidek RS 3000 advanced. FA, following fundus photography, patients underwent intravenous retinal fluorescein angiography. According to our standard technique, 5 mL of a 20% solution of sodium fluorescein was rapidly injected into the antecubital vein; then all phases of fluorescein transit in the posterior pole of the study eye were photographically recorded. Some late angiograms of the fellow eye were taken to confirm or exclude exudative AMD.

The following subject exclusion criteria were used: (i) unrelated eye disorders, for example, high refractive error, cloudy cornea, lens opacity (nuclear, cortical, or posterior subcapsular cataract) except minor opacities, keratitis, acute or chronic uveitis, glaucoma, or diseases of the optic nerve; (ii) systemic illnesses, for example, diabetes mellitus, malignant tumours, systemic connective tissue disorders, chronic infectious diseases, or conditions following organ or tissue transplantation; (iii) ungraded colour fundus photographs resulting from obscuration of the ocular optic system or because of fundus photograph quality.

### 2.3. DNA Extraction and Genotyping

The DNA extraction and analysis of the gene polymorphism of* CYP4F2 *(rs2108622) were carried out at the Laboratory of Ophthalmology at the Institute of Neuroscience of LUHS. DNA was extracted from 200 *μ*L venous blood (white blood cells) using a DNA purification kit based on the magnetic beads method (MagJET Genomic DNA Kit, Thermo Scientific) or the silica-based membrane technology utilizing a genomic DNA extraction kit (GeneJET Genomic DNA Purification Kit, Thermo Scientific), according to the manufacturer's recommendations.

The genotyping of* CYP4F2 *(rs2108622) was carried out using the real-time polymerase chain reaction (PCR) method. Single-nucleotide polymorphism was determined using TaqMan® Drug Metabolism assay (Thermo Scientific).

The genotyping was performed using a Rotor-Gene Q real-time PCR quantification system (Qiagen, USA). The real-time PCR reagents (2x TaqMan® Universal Master Mix, TaqMan® Drug Metabolism assay, nuclease-free water) were taken out from an environment of −20°C and were thawed at room temperature. The thawed reagents were centrifuged (10 000 rpm) and stored in an ice tub. Appropriate real-time PCR mixtures of* CYP4F2 *(rs2108622) were prepared for determining a single-nucleotide polymorphism.

A PCR reaction mixture (9 *μ*L) was poured into the 72 wells of the Rotor-Disc, and then 1 *μ*L of matrix DNA of the samples (~10 ng) and 1 *μ*L of negative control (−K) were added.

The Allelic Discrimination program was used during the real-time PCR. Then, the assay was continued following the manual provided by the manufacturer (http://www.qiagen.com/, Allelic Discrimination). After that, the Allelic Discrimination program was completed, and the genotyping results were received. The program determined the individual genotypes according to the fluorescence intensity rate of different detectors (VIC and FAM).

### 2.4. Statistical Analysis

Statistical analysis was performed using the SPSS/W 20.0 software (Statistical Package for the Social Sciences for Windows, Inc., Chicago, Illinois, USA). The data are presented as absolute numbers with percentages in brackets, average values, and standard deviations (SD). The frequencies of genotypes (in percentage) are presented in Table 2.

Hardy-Weinberg analysis was performed to compare the observed and expected frequencies of rs2108622 using the *χ* ^2^ test in all groups. The distribution of the rs2108622 single-nucleotide polymorphism (SNP) in the AMD and control groups was compared using the *χ* ^2^ test or the Fisher exact test. Binomial logistic regression analysis was performed to estimate the impact of genotypes on AMD development. Odds ratios and 95% confidence intervals are presented. The selection of the best genetic model was based on the Akaike Information Criterion (AIC); therefore, the best genetic models were those with the lowest AIC values.

Differences were considered statistically significant when *p* < 0.05.

## 3. Results

A total of 190 patients with early AMD and 181 patients with eAMD were enrolled into the analysis according to the subject inclusion and exclusion criteria. The control group comprised 210 persons. There were 76.7% (*n* = 161) of women in the control group, 70% (*n* = 133) of women in the early AMD group, and 67.4% (*n* = 122) of women in the eAMD group.

The genotyping of rs2108622 was performed in patients with early AMD and eAMD and in the control group subjects (Tables 2 and 3).

The distribution of the analyzed rs2108622 genotype and allele frequencies in early AMD patients, eAMD patients, and controls matched the Hardy-Weinberg equilibrium. rs2108622 gene polymorphism analysis in the overall group did not reveal any differences in the genotypes distribution between patients with AMD and control group subjects.

The comparison of the rs2108622 genotype frequency by age groups did not reveal significant differences as well (Table 4).

The comparison of the rs2108622 genotype in males and females between patients with early AMD and the control group did not show statistically significant differences but revealed statistically significant differences between males and females with eAMD. The* CYP4F2 *(1347C>T)* T/T* genotype was more frequent in males with eAMD (10.2% versus 0.8%; *p* = 0.0052). When comparing the* CYP4F2 *genotype distribution in females with eAMD and healthy females significant differences were also revealed. The* CYP4F2 *(1347C>T)* T/T *genotype was less frequently present in eAMD females compared to healthy controls (0.8% versus 6.2%; *p* = 0.027) (Table 5).

Binomial logistic regression analysis in the patients with NeAMD and in the control group was performed (Table 6). Similarly, binomial logistic regression analysis in the patients with eAMD and in the control group was carried out (Table 7). These analyses did not detect any differences in the models between patients with AMD and control group subjects.

Binomial logistic regression analysis in males and females with AMD and in the control group was performed (Table 8). In the female AMD group this analysis revealed that the recessive (*p* value = 0.049) variables were statistically significant (Table 8).

Binomial logistic regression analysis in the males with early AMD and in the control group by age was performed (Table 9). This analysis found that the additive (*p* = 0.041) variables were statistically significant in the group aged over 65 years.

Binomial logistic regression analysis in the males with eAMD and in the control group by age was performed (Table 10). This analysis displayed that the codominant (*p* = 0.026) variables in the group under 65 years old were statistically significant (Table 10). Moreover, the dominant (*p* = 0.037) and additive (*p* = 0.015) variables were statistically significant in the group of over 65 years (Table 10).

## 4. Discussion

It is known that changes in the metabolism of cholesterol, particularly of HDL, may influence drusen accumulation and consequently promote AMD development [17]. Cholesterol can be obtained from systemic sources or recycled in the retina, through the circulation of blood lipids. Thus, the retinal rod cells are capable of using both lipids from the liver and those recycled from RPE [18]. With advancing age, there is a deposit of lipid particles in a normal BrM leading to the creation of a lipid wall, external to the RPE basal lamina, impairing nutrient exchange between the choriocapillaris and the RPE which compromises retinal function; thus that accumulation of lipids seems to play a pivotal role in the development of AMD [6, 7, 10]. In addition to genetic predisposition, which accounts for 70% of the risk of the disease development [19], advanced age is also considered a risk factor, which results in the deposition of lipid particles and formation of drusen in the retina, thereby affecting retinal function [18]. The study done by Björkhem et al. states that the most important enzymatic oxidation of cholesterol takes place with the involvement of the* CYP46A1* and* CYP27A1 *loci of cytochrome P450 family which forms 24-hydroxycholesterol (24-Hch) and 27-hydroxycholesterol (27-Hch), respectively [19]. High levels of 27-Kch have been reported in macrophages foam cells and in atherosclerotic plaque but have not been documented in the retina [20, 21]. These population-based genetic studies coupled with animal experiments indicate the prominent role of cholesterol metabolism in the AMD pathology [20, 21]. The rs754203 SNP in the* CYP46A1* gene was evaluated by Fourgeux et al. and no significant difference between the* CYP46A1* genotypes in the AMD and control groups was observed [22]. At the same time, the data from three studies are controversial, due to conflicting results. Therefore, we aimed to determine the frequency of the genotype of 1347C>T (rs2108622) in* CYP4F2* (which is involved in lipid metabolism) in patients with early and exudative AMD. The data of our analysis of rs2108622 polymorphism did not reveal any differences in the distribution of C/C, C/T, and T/T genotypes between the early AMD group, exudative AMD group, and the control group (51.6%, 42.6%, and 5.8% in early AMD group, 56.35%, 39.78%, and 3.87% in the exudative AMD group, and 53.33%, 39.05%, and 7.62%, in the control group, resp.). The* CYP4F2 *(1347C>T)* T/T* genotype was more frequent in males with exudative AMD (10.2% versus 0.8%; *p* = 0.0052), and the comparison of the* CYP4F2 *genotype distribution in females with exudative AMD and healthy females determined significant differences. The* CYP4F2 *(1347C>T)* T/T *genotype was less frequently present in exudative AMD females compared to healthy control females (0.8% versus 6.2%; *p* = 0.027). To interpret our results, the research should be repeated with a larger sample size. However, exposure to environmental hazards has been associated with diseases in humans. The identification of single-nucleotide polymorphism (SNP) in human populations exposed to different environmental hazards is vital for detecting the genetic risks of some important human diseases [23]. Yang et al. study sought to structure a genetic score for smoking behaviour in a Chinese population. They tested GWAS-significant SNPs associated with smoking behaviour in a Chinese population and structured three types of genetic scores. They found that the effects of the three types of genetic score were similar; however, to best extrapolate and understand these types of results, the unweighted genetic score represents the ideal choice. Furthermore, the genetic score was significantly associated with smoking behaviour (smoking status and SI at ≤18 years of age). The results of this study may guide relevant health education for those with a high genetic score and promote smoking control to improve the health of the population [24]. It is possible that the genotype distribution of rs2108622 in the patients with exudative age-related macular degeneration (AMD) and the control subjects by gender can result from gene-environment interactions. One of the environmental risk factors could be smoking.

We found no studies analyzing the* CYP4F2 *(1347C>T, rs2108622) gene polymorphism in patients with age-related macular degeneration; thus we can only compare the genotyping results in our control group with results obtained in other studies.

Results of the control groups with rs2108622 polymorphism distribution in the other studies are shown in Table 11. It shows quite similar results to our study except for one study in Saudi Arabia [25] but it remains unclear if the reason for this is ethnicity, the size of control group, or something else.

There are many studies analyzing genes that take part in lipid metabolism and about systemic lipoproteins, but results are conflicting. Moreover, conflicting results have been reported with regard to the associations of AMD with serum HDL concentration and the genes involved in lipid metabolism. Some authors agree that increased serum cholesterol levels increase AMD development [16, 26]; however, other authors disagree [27] and some others reported no significant associations [28–31]. Ebrahimi and Handa suggested that before excluding the role of systemic lipids in AMD, the role of plasma lipids in the context of genotype could be examined to identify predisposition in a subset of patients at risk of developing AMD due to genotype and plasma lipid levels [32]. As the major SNPs involved in the HDL pathway, CETP rs3764261, LPL rs12678919, and LIPC rs10468017 have been shown to be associated with AMD in a genome-wide association studies (GWAS) [33]. However, previous studies on the reported HDL cholesterol metabolism genes showed contradictory results, especially in several GWAS; moreover, their genetic susceptibility to AMD varied in diverse populations [34–36]. On the other hand, different studies have found an inverse correlation with HDL and AMD risk. Reynolds et al. [37] recently demonstrated that elevated HDL is associated with a reduced risk of advanced AMD, especially the neovascular (NV) subtype (*p* value < 0.05, 0.03, resp.), and that higher low density lipoprotein (LDL) is associated with an increased risk of advanced AMD and the NV subtype (*p* value < 0.03, 0.04, resp.). When looking for an association of serum lipids with advanced AMD in multivariate modeling, Reynolds et al. also found significant trends with a higher quartile of LDL and increasing AMD risk (*p* for trend <0.03, 0.01). Higher total cholesterol was also associated with AMD risk when controlling for all covariates and genotypes [37].

A Klein et al. study [38] examined 6950 participants from the Beaver Dam Eye Study (BDES), Blue Mountains Eye Study (BMES), and Rotterdam Study (RS). There were few associations among total cholesterol, HDL-C, total cholesterol/HDL-C ratio, and non-HDL-C and the incidence of early AMD, soft indistinct (SI) drusen, large area of drusen, pigmentary abnormalities, late AMD, and exudative AMD. Direct associations of HDL-C with the incidence of pure geographic atrophy (GA) were present in the BDES and RS cohorts but not the BMES cohort. The use of statins was not associated with any incidence of an AMD outcome in any of the cohorts. In the BDES,* CETP* rs1864163 and* LPL* rs281 were protective against the development of large drusen area in the macula and* CETP* rs3764261 was protective against the development of pure GA. In the RS,* CETP* rs3764261 was linked to an increased risk of late AMD, pure GA, and exudative AMD.* CETP* rs1864163 was related to a decreased risk of the incidence of early AMD and large drusen area.* ABCA1* rs1883025 was associated with a decreased risk of large SI drusen.* LPL* rs281 was associated with a decreased risk of a large drusen area in the macula. In the BMES, none of these SNPs were found to be significantly associated with any AMD outcomes, although marginally nonsignificant associations were found between* ABCA1* rs1883025/T and pure GA and between* LIPC* rs7163555 and exudative AMD. Mean HDL-C increased in each of the three cohorts with each additional risk allele for* CETP* rs376426 and decreased with each additional risk allele for* CETP* rs1864163 and* ABCA1* rs1883025 (all *p* < 0.05). There were no consistent statistically significant associations between* LIPC* or* LPL* and HDL-C or between any of the lipid genes and total cholesterol [38]. In a meta-analysis, after correction for multiple testing, they did not find an association between serum lipids or lipid pathway genes and the incidence or progression of AMD over a 20-year period using data from three population-based cohort studies, the BDES, BMES, and RS [38].

In addition to being one of the major AMD-susceptibility genes, perhaps accounting for approximately 30%–50% of AMD patients, CFH might interact with lipid metabolism to affect the disease risk [39]. Such interactions among genes in the complement system and HDL metabolism pathway might be proposed as one potential explanation for the lack of heritability [40]. However, whether the interaction is present or not and whether or not these interactions occur with different genes in the HDL metabolism pathway remain unclear [40].

In conclusion, our results suggest that the CYP4F2 (rs2108622) gene polymorphism had no predominant effect on the development of early and eAMD when compared to the control group. But the* CYP4F2 *(1347C>T)* T/T* genotype was more frequent in males with eAMD compared to females (10.2% versus 0.8%; *p* = 0.0052), and the comparison of the* CYP4F2 *genotype distribution in females with eAMD and healthy females did not reveal significant difference. The* CYP4F2 *(1347C>T)* T/T *genotype was less frequently present in eAMD females compared to healthy control females (0.8% versus 6.2%; *p* = 0.027), but this must be replicated with a larger sample size to prove these results, as we know that T/T genotype is wild and very rare.

## Figures and Tables

**Table 1:** Demographic characteristics of the study population.

Characteristic	Group			*p* value
	Early AMD *n* = 190	eAMD *n* = 181	Control *n* = 210	
Men, *n* (%)	57 (30.0)	59 (32.6)	49 (23.3)	NS
Women, *n* (%)	133 (70.0)	122 (67.4)	161 (76.7)	NS
Age median (min; max)	66 (50; 93)	76 (50; 90)	52 (20; 90)	NS

NS: nonsignificant.

**Table 2:** Frequency of the CYP4F2 rs2108622 genotypes in the patients with early AMD and in the control group.

Genotype/allele	Frequency (%)				
	Control group *n* (%) (*n* = 210)	*p* value HWE	Early AMD group *n* (%) (*n* = 190)	*p* value HWE	*p* value
Genotype		0.854		0.277	
T/T	16 (7.62)		11 (5.8)		*χ* ^2^ = 0.8676 *p* = 0.6481
T/C	82 (39.05)		81 (42.6)		
C/C	112 (53.33)		98 (51.6)		
*Total*	*210 (100)*		*190 (100)*		
Allele					
T	114 (25.33)		103 (31.21)		
C	336 (74.67)		277 (68.79)		

*p* value: significance level (alpha = 0.05); *p* value HWE: significance level (alpha = 0.05) by Hardy-Weinberg equilibrium.

**Table 3:** Frequency of the CYP4F2 rs2108622 genotypes in the patients with eAMD and in the control group.

Genotype/allele	Frequency (%)				
	Control group *n* (%) (*n* = 210)	*p* value HWE	eAMD group *n* (%) (*n* = 181)	*p* value HWE	*p* value
Genotype		0.854		0.187	
T/T	16 (7.62)		7 (3.87)		*χ* ^2^ = 2.5012 *p* = 0.2863
T/C	82 (39.05)		72 (39.78)		
C/C	112 (53.33)		102 (56.35)		
*Total*	*210 T (1000)*		*181 (100)*		
Allele					
T	114 (25.33)		86 (23.75)		
C	336 (74.67)		276 (76.25)		

*p* value: significance level (alpha = 0.05); *p* value HWE: significance level (alpha = 0.05) by Hardy-Weinberg equilibrium.

**Table 4:** Frequency of *CYP4F2* rs2108622 genotype in early AMD patients, in eAMD patients and controls by age.

Genotype	<65 years		*p* value	≥65 years		*p* value
	AMD group *n* (%)	Control group *n* (%)		AMD group *n* (%)	Control group *n* (%)	
Frequency of *CYP4F2 (1347C>T; V433M) rs2108622* genotype in the patients with early AMD and the control subjects by age						
TT	4 (4.5)	11 (7.1)	0.538	7 (6.9)	5 (9.3)	0.847
TC	39 (43.8)	59 (37.8)		42 (41.6)	23 (42.6)	
CC	46 (51.7)	86 (55.1)		52 (51.5)	26 (48.1)	
Allele						
T	47 (26.4)	81 (25.96)		56 (27.72)	33 (30.55)	
C	131 (73.6)	231 (74.04)		146 (72.28)	75 (69.45)	

Frequency of *CYP4F2 (1347C>T; V433M) rs2108622* genotype in the patients with eAMD and the control subjects by age						
TT	1 (3.8)	11 (7.1)	0.751	6 (3.9)	5 (9.3)	0.266
TC	9 (34.6)	59 (37.8)		63 (40.6)	23 (42.6)	
CC	16 (61.5)	86 (55.1)		86 (55.5)	26 (48.1)	
Allele						
T	11 (21.15)	81 (25.96)		75 (24.19)	33 (30.55)	
C	41 (78.85)	231 (74.04)		235 (75.81)	75 (69.45)	

*p* value: significance level (alpha = 0.05); *p* value HWE: significance level (alpha = 0.05) by Hardy-Weinberg equilibrium.

**Table 5:** Frequency of CYP4F2 rs2108622 genotype in the patients with early AMD and eAMD and the control subjects by gender.

Genotype	Males		*p* value	Females		*p* value
	AMD group *n* (%)	Control group *n* (%)		AMD group *n* (%)	Control group *n* (%)	
Frequency of *CYP4F2 (1347C>T; V433M) rs2108622* genotype in the patients with early age-related macular degeneration (AMD) and the control subjects by gender						
TT	6 (10.5)	6 (12.2)	0.149	5 (3.8)	10 (6.2)	0.429
TC	27 (47.4)	23 (46.9)	1	54 (40.6)	59 (36.6)	0.547
CC	24 (42.1)	20 (40.8)	1	74 (55.6)	92 (57.1)	0.814
Allele						
T	39 (34.51)	35 (35.71)		64 (24.06)	79 (24.53)	
C	75 (65.49)	63 (64.29)		202 (75.94)	243 (75.47)	

Frequency of *CYP4F2 (1347C>T; V433M) rs2108622* genotype in the patients with exudative age-related macular degeneration (AMD) and the control subjects by gender						
TT	*6 (10.2)* ^*∗*^	6 (12.2)	0.767	*1 (0.8)* ^*∗*^	*10 (6.2)*	*0.027*
TC	22 (37.7)	23 (46.9)	0.333	50 (41.0)	59 (36.6)	0.462
CC	31 (52.5)	20 (40.8)	0.250	71 (58.2)	92 (57.1)	0.904
Allele						
T	34 (28.81)	35 (35.71)		50 (20.66)	79 (24.53)	
C	84 (71.19)	63 (64.29)		192 (79.34)	243 (75.47)	

^*∗*^ *p* = 0.0052.

*p* value: significance level (alpha = 0.05); *p* value HWE: significance level (alpha = 0.05) by Hardy-Weinberg equilibrium.

**Table 6:** Binomial logistic regression analysis of the *CYP4F2 (1347C>T; V433M) rs2108622* in the patients with early AMD and in the control group.

Model	Genotype	OR (95% CI)	*p* value	AIC
Codominant	T/T	0.786 (0.348; 1.774)	0.562	558.646
	T/C	1.129 (0.750; 1.700)	0.562	
Dominant	T/C + T/T	1.073 (0.724; 1.589)	0.726	557.394
Recessive	T/T	0.745 (0.337; 1.649)	0.468	556.983
Overdominant	T/C	1.160 (0.778; 1.729)	0.466	556.987
Additive	—	1.002 (0.730; 1.376)	0.990	557.517

**Table 7:** Binomial logistic regression analysis of the *CYP4F2 (1347C>T; V433M) rs2108622* in the patients with eAMD and in the control group.

Model	Genotype	OR (95% CI)	*p* value	AIC
Codominant	T/T	0.480 (0.190; 1.215)	0.121	543.306
	T/C	0.964 (0.637; 1.460)	0.863	
Dominant	T/C + T/T	0.885 (0.593; 1.320)	0.550	543.530
Recessive	T/T	0.488 (0.196; 1.214)	0.123	541.336
Overdominant	T/C	1.031 (0.687; 1.549)	0.883	543.866
Additive	—	1.204 (0.865; 1.675)	0.271	542.671

**Table 8:** Binomial logistic regression analysis of the *CYP4F2 (1347C>T; V433M) rs2108622* in males and females with eAMD and in the control group.

Model	Genotype	OR (95% CI)	*p* value	AIC
Female				
Codominant	T/T	0.130 (0.016; 1.036)	0.054	386.317
	T/C	1.098 (0.674; 1.788)	0.707	
Dominant	T/C + T/T	0.958 (0.595; 1.542)	0.859	390.898
*Recessive*	*T/T*	*0.125 (0.016; 0.988)*	*0.049*	*384.458*
Overdominant	T/C	1.201 (0.741; 1.945)	0.458	390.379
Additive	—	1.220 (0.805; 1.850)	0.348	390.041
Male				
Codominant	T/T	0.645 (0.182; 2.282)	0.497	153.306
	T/C	0.617 (0.274; 1.389)	0.243	
Dominant	T/C + T/T	0.623 (0.290; 1.339)	0.225	151.311
Recessive	T/T	0.811 (0.244; 2.696)	0.733	152.676
Overdominant	T/C	0.672 (0.311; 1.452)	0.312	151.767
Additive	—	1.357 (0.770; 2.392)	0.291	151.666

**Table 9:** Binomial logistic regression analysis of the *CYP4F2 (1347C>T; V433M) rs2108622* in the males with early AMD and in the control group by age.

Model	Genotype	OR (95% CI)	*p* value	AIC
<65				
Codominant	T/T	1.727 (0.296; 10.082)	0.544	97.984
	T/C	1.524 (0.551; 4.212)	0.417	
Dominant	T/C + T/T	1.555 (0.584; 4.135)	0.377	96.003
Recessive	T/T	1.385 (0.259; 7.414)	0.704	96.648
Overdominant	T/C	1.387 (0.528; 3.639)	0.507	96.350
Additive	—	0.717 (0.335; 1.533)	0.391	96.050
≥65				
Codominant	T/T	0.077 (0.006; 1.023)	0.052	44.437
	T/C	0.154 (0.016; 1.471)	0.104	
Dominant	T/C + T/T	0.128 (0.014; 1.152)	0.067	42.960
Recessive	T/T	0.280 (0.046; 1.705)	0.167	45.938
Overdominant	T/C	0.500 (0.115; 2.175)	0.355	46.931
*Additive*	—	3.422 (1.052; 11.130)	*0.041*	42.961

**Table 10:** Binomial logistic regression analysis of the *CYP4F2 (1347C>T; V433M) rs2108622* in the males with eAMD and in the control group by age.

Model	Genotype	OR (95% CI)	*p* value	AIC
<65				
Codominant	T/T	1.583 (0.129; 19.422)	0.719	44.539
	T/C	0.559 (0.091; 3.446)	0.531	
Dominant	T/C + T/T	0.713 (0.141; 3.612)	0.682	43.065
Recessive	T/T	2.00 (0.177; 22.550)	0.575	42.948
Overdominant	T/C	0.518 (0.089; 3.002)	0.463	42.662
Additive	—	1.045 (0.298; 3.667)	0.945	43.230
≥65				
Codominant	T/T	0.062 (0.005; 0.720)	*0.026*	53.304
	T/C	0.123 (0.014; 1.108)	0.062	
*Dominant*	T/C + T/T	0.103 (0.012; 0.871)	*0.037*	51.927
Recessive	T/T	0.248 (0.048; 1.276)	0.095	56.239
Overdominant	T/C	0.417 (0.105; 1.661)	0.215	57.207
*Additive*	—	3.670 (1.282; 10.502)	*0.015*	52.149

**Table 11:** Frequency of CYP4F2 rs2108622 genotypes in the control groups from other studies.

	Study	Country	VV%	VM%	MM%	Total
1	Our study	Lithuania	53.33	39.05	7.62	
2	Ivashchenko et al. 2013 [41]	Russia	57.1	34.1	7.7	—
3	Cen et al. 2010 [42]	China	52.0	41.0	7.0	—
4	Teichert et al. 2009 [43]	Caucasian	54.4	37.1	8.4	—
5	Zeng et al. 2012 [44]	China	55.1	40.5	4.4	370

## References

[1] Lim L. S., Mitchell P., Seddon J. M., Holz F. G., Wong T. Y. (2012). Age-related macular degeneration. *The Lancet*.

[2] Jager R. D., Mieler W. F., Miller J. W. (2008). Age-related macular degeneration. *The New England Journal of Medicine*.

[3] Wang Y.-F., Han Y., Zhang R., Qin L., Wang M.-X., Ma L. (2015). CETP/LPL/LIPC gene polymorphisms and susceptibility to age-related macular degeneration. *Scientific Reports*.

[4] Young R. W. (1987). Pathophysiology of age-related macular degeneration. *Survey of Ophthalmology*.

[5] Zarbin M. A. (2004). Current concepts in the pathogenesis of age-related macular degeneration. *Archives of Ophthalmology*.

[6] Loebstein R., Yonath H., Peleg D. (2001). Pharmacoepidemiology and drug utilization: interindividual variability in sensitivity to warfarin—nature or nurture?. *Clinical Pharmacology and Therapeutics*.

[7] Taube J., Halsall D., Baglin T. (2000). Influence of cytochrome P-450 CYP2C9 polymorphisms on warfarin sensitivity and risk of over-anticoagulation in patients on long-term treatment. *Blood*.

[8] D'Andrea G., D'Ambrosio R. L., Di Perna P. (2005). A polymorphism in the VKORC1 gene is associated with an interindividual variability in the dose-anticoagulant effect of warfarin. *Blood*.

[9] Caldwell M. D., Awad T., Johnson J. A. (2008). CYK4F-2 genetic variant alters required warfarin dose. *Blood*.

[10] Koskela A., Reinisalo M., Hyttinen J. M. T., Kaarniranta K., Karjalainen R. O. (2014). Pinosylvin-mediated protection against oxidative stress in human retinal pigment epithelial cells. *Molecular Vision*.

[11] Lai G., Wu J., Liu X., Zhao Y. (2012). 20-HETE induces hyperglycemia through the cAMP/ PKA-PhK-GP pathway. *Molecular Endocrinology*.

[12] Theken K. N., Deng Y., Alison Kannon M., Miller T. M., Poloyac S. M., Lee C. R. (2011). Activation of the acute inflammatory response alters cytochrome P450 expression and eicosanoid metabolism. *Drug Metabolism and Disposition*.

[13] Hardwick J. P. (2008). Cytochrome P450 omega hydroxylase (CYP4) function in fatty acid metabolism and metabolic diseases. *Biochemical Pharmacology*.

[14] Fava C., Montagnana M., Danese E. (2012). The functional variant V433M of the CYP4F2 and the metabolic syndrome in Swedes. *Prostaglandins and Other Lipid Mediators*.

[15] Age-Related Eye Disease Study Research Group (2001). The age-related eye disease study system for classifying age-related macular degeneration from stereoscopic color fundus photographs: the age-related eye disease study report number. *American Journal of Ophthalmology*.

[16] Spencer K. L., Olson L. M., Schnetz-Boutaud N. (2011). Using genetic variation and environmental risk factor data to identify individuals at high risk for age-related macular degeneration. *PLoS ONE*.

[17] Cezario S. M., Calastri M. C., Oliveira C. I. (2015). Association of high-density lipoprotein and apolipoprotein E genetic variants with age-related maculardegeneration. *Arquivos Brasileiros de Oftalmologia*.

[18] Curcio C. A., Johnson M., Rudolf M., Huang J.-D. (2011). The oil spill in ageing Bruch membrane. *British Journal of Ophthalmology*.

[19] Björkhem I., Cedazo-Minguez A., Leoni V., Meaney S. (2009). Oxysterols and neurodegenerative diseases. *Molecular Aspects of Medicine*.

[20] Björkhem I., Andersson O., Diczfalusy U. (1994). Atherosclerosis and sterol 27-hydroxylase: evidence for a role of this enzyme in elimination of cholesterol from human macrophages. *Proceedings of the National Academy of Sciences of the United States of America*.

[21] Luoma P. V. (2008). Cytochrome P450 and gene activation—from pharmacology to cholesterol elimination and regression of atherosclerosis. *European Journal of Clinical Pharmacology*.

[22] Fourgeux C., Dugas B., Richard F. (2012). Single nucleotide polymorphism in the cholesterol-24SHydroxylase (CYP46A1) hene and Its association with CFH and LOC387715 gene polymorphisms in age-related macular degeneration. *Investigative Ophthalmology and Visual Science*.

[23] Hollman A. L., Tchounwou P. B., Huang H. C. (2016). The Association between gene-environment interactions and diseases involving the human GST superfamily with SNP variants. *International Journal of Environmental Research and Public Health*.

[24] Yang S., He Y., Wang J. (2016). Genetic scores of smoking behaviour in a Chinese population. *Scientific Reports*.

[25] Munshi A., Sharma V., Kaul S. (2012). Association of 1347 G/A cytochrome P450 4F2 (CYP4F2) gene variant with hypertension and stroke. *Molecular Biology Reports*.

[26] Cougnard-Grégoire A., Delyfer M.-N., Korobelnik J.-F. (2014). Elevated high-density lipoprotein cholesterol and age-related macular degeneration: the Alienor study. *PLoS ONE*.

[27] Chakravarthy U., Wong T. Y., Fletcher A. (2010). Clinical risk factors for age-related macular degeneration: a systematic review and meta-analysis. *BMC Ophthalmology*.

[28] Ulas F., Balbaba M., Ozmen S., Celebi S., Dogan U. (2013). Association of dehydroepiandrosterone sulfate, serum lipids, C-reactive protein and body mass index with age-related macular degeneration. *International Ophthalmology*.

[29] Cackett P., Wong T. Y., Aung T. (2008). Smoking, cardiovascular risk factors, and age-related macular degeneration in asians: the singapore malay eye study. *American Journal of Ophthalmology*.

[30] Smith W., Mitchell P., Leeder S. R., Wang J. J. (1998). Plasma fibrinogen levels, other cardiovascular risk factors, and age-related maculopathy: the Blue Mountains Eye Study. *Archives of Ophthalmology*.

[31] Tomany S. C., Wang J. J., Van Leeuwen R. (2004). Risk factors for incident age-related macular degeneration: pooled findings from 3 continents. *Ophthalmology*.

[32] Ebrahimi K. B., Handa J. T. (2011). Lipids, lipoproteins, and age-related macular degeneration. *Journal of Lipids*.

[33] Neale B. M., Fagerness J., Reynolds R. (2010). Genome-wide association study of advanced age-related macular degeneration identifies a role of the hepatic lipase gene (LIPC). *Proceedings of the National Academy of Sciences of the United States of America*.

[34] Cipriani V., Leung H.-T., Plagnol V. (2012). Genome-wide association study of age-related macular degeneration identifies associated variants in the TNXB-FKBPL-NOTCH4 region of chromosome 6p21.3. *Human Molecular Genetics*.

[35] Chen W., Stambolian D., Edwards A. O. (2010). Genetic variants near TIMP3 and high-density lipoprotein-associated loci influence susceptibility to age-related macular degeneration. *Proceedings of the National Academy of Sciences of the United States of America*.

[36] Restrepo N. A., Spencer K. L., Goodloe R. (2014). Genetic determinants of age-related macular degeneration in diverse populations from the PAGE study. *Investigative Ophthalmology & Visual Science*.

[37] Reynolds R., Rosner B., Seddon J. M. (2010). Serum lipid biomarkers and hepatic lipase gene associations with age-related macular degeneration. *Ophthalmology*.

[38] Klein R., Myers C. E., Buitendijk G. H. S. (2014). Lipids, lipid genes, and incident age-related macular degeneration: the three continent age-related macular degeneration consortium. *American Journal of Ophthalmology*.

[39] Haines J. L., Hauser M. A., Schmidt S. (2005). Complement factor H variant increases the risk of age-related macular degeneration. *Science*.

[40] Zuk O., Hechter E., Sunyaev S. R., Lander E. S. (2012). The mystery of missing heritability: genetic interactions create phantom heritability. *Proceedings of the National Academy of Sciences of the United States of America*.

[41] Ivashchenko D., Rusin I., Sychev D., Grachev A. (2013). The frequency of CYP2C9, VKORC1, and CYP4F2 polymorphisms in Russian patients with high thrombotic risk. *Medicina*.

[42] Cen H.-J., Zeng W.-T., Leng X.-Y. (2010). *CYP4F2* rs2108622: a minor significant genetic factor of warfarin dose in Han Chinese patients with mechanical heart valve replacement. *British Journal of Clinical Pharmacology*.

[43] Teichert M., Eijgelsheim M., Rivadeneira F. (2009). A genome-wide association study of acenocoumarol maintenance dosage. *Human Molecular Genetics*.

[44] Zeng W. T., Zheng Q. S., Huang M. (2012). Genetic polymorphisms of VKORC1, CYP2C9, CYP4F2 in Bai, Tibetan Chinese. *Pharmazie*.

